# Implantable Cardioverter‐Defibrillators in Children: A 14‐Year Population‐Based Study

**DOI:** 10.1002/joa3.70308

**Published:** 2026-03-08

**Authors:** Marko Bjeloševič, Peter Olejník, Jaroslav Tomko, Michal Chalupka, Viera Illíková

**Affiliations:** ^1^ Department of Paediatric Cardiology, Faculty of Medicine Comenius University in Bratislava Bratislava Slovakia; ^2^ Department of Arrhythmias and Cardiac Pacing Paediatric Cardiac Centre, National Institute of Cardiovascular Diseases in Bratislava Bratislava Slovakia; ^3^ Department of Paediatric Cardiology Paediatric Cardiac Centre, National Institute of Cardiovascular Diseases in Bratislava Bratislava Slovakia

**Keywords:** EV‐ICD, ICD, pediatric, S‐ICD

## Abstract

**Aims:**

Implantable cardioverter‐defibrillator (ICD) therapy in children, particularly for primary prevention, remains under investigation, with limited data from less affluent European countries. With increasing use of fully subcutaneous (S‐ICD) and extravascular (EV‐ICD) systems, we analyzed epidemiology, indications, complications, and outcomes in pediatric ICD recipients at our tertiary center.

**Methods:**

This retrospective, population‐based, nationwide, single‐center study consecutively analyzed medical records from 2012 to 2025. Patients aged 0–18 years who underwent ICD implantation were included. Kaplan–Meier analyses were performed for shock‐free and mortality endpoints.

**Results:**

Forty‐nine patients were included, corresponding to an estimated national incidence of approximately one ICD implantation per 22 000 live births. ICDs were predominantly implanted for secondary prevention (80%). Long QT syndrome (32%) and hypertrophic cardiomyopathy (22%) were the most frequent diagnoses. During 207 patient‐years of follow‐up (median follow‐up 4 years), appropriate ICD shocks occurred in 27% of patients exclusively in the secondary prevention group. No appropriate therapies occurred in the primary prevention group despite higher overall mortality. Inappropriate shocks occurred in 22% of patients. Device‐related major complications affected 17% of implanted systems. Median transvenous ICD battery longevity was 6.5 years. Overall mortality was 6%, all related to underlying structural heart disease, with no ICD‐related deaths. S‐ICDs demonstrated a favorable acute and mid‐term safety profile in patients without pacing requirements.

**Conclusions:**

Pediatric ICD therapy is limited by substantial device‐related morbidity and imperfect risk stratification for primary prevention. The emerging S‐ICD and EV‐ICD systems represent promising, less invasive alternatives for selected pediatric patients.

## Graphical abstract

Nationwide pediatric ICD cohort from Slovakia illustrating incidence, indications, shock outcomes, and device‐related adverse events, highlighting the predominance of secondary prevention and the clinical burden of inappropriate shocks and major complications.

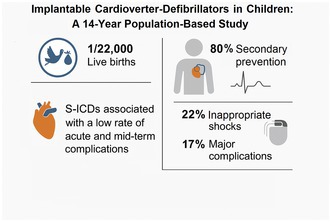

## 1 Introduction

Implantable cardioverter‐defibrillator (ICD) therapy in children, particularly for primary prevention, remains an area of ongoing research and clinical interest. However, most recent data originate from high gross domestic product (GDP) countries, with limited representation from those with lower GDPs [1, 2, 3, 4]. In fact, only two European publications have emerged from such settings over the past decade [5, 6]. To contribute to this underrepresented area of knowledge, we present a retrospective study analyzing the epidemiology, indications, complications, device longevity, and mortality in children who underwent ICD implantation over a 14‐year period at a single nationwide center.

## 2 Material and Methods

### 2.1 Study Design

This retrospective, population‐based, nationwide, single‐center study analyzed various aspects of ICD therapy in a consecutive cohort of 49 patients implanted over a 14‐year period, from 1 January 2012 to 1 October 2025.

Slovakia operates a highly centralized national healthcare system for pediatric arrhythmias. All patients younger than 18 years of age requiring specialist arrhythmia management, including ICD implantation, are referred to a single tertiary center—the Pediatric Cardiac Center of the National Institute of Cardiovascular Diseases in Bratislava—which serves as the sole national referral unit for pediatric arrhythmia management in Slovakia. In accordance with established national clinical practice pathways, patients aged exactly 18 years are also very likely to be referred to this center; however, a minimal degree of overlap with adult healthcare facilities cannot be entirely excluded.

#### 2.1.1 Inclusion Criteria

- Patients who received an ICD implantation at our center between 0 and 18 years of age and were followed up between 1 January 2012 and 1 October 2025 (~14 years).

#### 2.1.2 Exclusion Criteria

- Patients implanted with an ICD at our pediatric center at an age > 18 years (*N* = 2) were excluded to avoid overlap with adult‐care facilities.

Data were obtained from institutional clinical records, including the ICD database and the electronic health record system. All patients were included using fully anonymized data. Identifiable personal information was removed prior to analysis, and no identifiable data were available to the investigators for the purposes of this study.

### 2.2 Statistical Analysis

Continuous data were presented as median and range, and categorical variables as counts and percentages. Time‐to‐event outcomes for appropriate shocks, inappropriate shocks, and all‐cause mortality were analyzed using Kaplan–Meier survival estimation, with follow‐up calculated from ICD implantation to the first occurrence of each endpoint or censoring. Survival curves for primary and secondary prevention groups were compared using the log‐rank test, with *p*‐values displayed on the corresponding figures (*p* < 0.05 considered statistically significant). Numbers of patients at risk were presented at predefined time intervals. Statistical analyses and figure generation were performed using Python‐based survival analysis methods. Statistical code development and figure formatting were assisted by ChatGPT (version 5.2; OpenAI). All AI‐generated statistical code was independently validated by rerunning the analyses in R (version 4.5.2) by human investigators, and all outputs were critically reviewed and verified by the authors.

### 2.3 Complication Classification and Reporting

ICD‐related complications were classified using a framework informed by previously published ICD lead studies, including Atallah et al. and the PLEASE study [7]. Complications were categorized as major or minor, and as early or late, according to the criteria below:

- Major complications were defined as events requiring surgical revision, device or lead extraction, lead replacement, or pocket revision, as well as those associated with clinically significant morbidity, such as device infection requiring prolonged antibiotic therapy or a lead‐associated thrombus requiring anticoagulation.
- Minor complications were defined as events not requiring surgical intervention, such as pocket hematoma managed conservatively, mild lead dislodgement corrected by device reprogramming, or superficial wound issues not requiring surgery or long‐term antibiotic therapy.
- Early (procedural) complications were defined as events occurring within 30 days of implantation, consistent with commonly used early/late classifications in ICD complication studies.
- Late complications were defined as events occurring after 30 days.

All complication rates are reported per device, unless otherwise specified, following the device‐based reporting convention used in multiple ICD lead performance studies, including the registry cohort evaluated by Atallah et al. [7].

### 2.4 Ethics Statement

The Ethics Committee of the National Institute of Cardiovascular Diseases reviewed the study and the methods used, and issued a waiver confirming that, in the context of a retrospective study using anonymized data, informed consent from participants was not required.

## 3 Results

During the study period of 14 years, 53 ICD devices were implanted in 49 patients (male 59%; 207 patient‐years; Table 1). The median follow‐up period was 4 years (1 month to 14 years).

**TABLE 1:** Device type, follow‐up, structural heart disease, shocks, and complications.

ICD type	Transvenous single‐chamber	Transvenous dual‐chamber	Fully subcutaneous	Hybrid^a^	Extravascular	Total
N of devices	25	6	18	2	2	53
N of patients	24	6	18	2	2	49^b^
Patient‐years	127.3	21.7	44.7	12.3	1.2	207.2
Median follow‐up	6 y (0.5–9)	3.5 y (0.7–6.9)	2.1 y (0.1–5.5)	6.2 y (1.3–11.1)	0.6 y (0.4–0.8)	4 y (0.1–14)
*N* of patients with structural heart disease	7 (29%)	6 (100%)	7 (39%)	0 (0%)	1 (50%)	21 (43%)
*N* of patients with appropriate shocks	10	0	3	1	0	13^c^ (27%)
*N* of appropriate shocks	33	0	3	13	0	49
*N* of patients with inappropriate shocks	5	1	4	1	0	11 (22%)
*N* of inappropriate shocks	14	4	8	8	0	34
Inappropriate shock cause	SVT, TWO, lead fracture	SVT	SVT, TWO, myopotentials, header air bubbles	SVT		
Complications	5 major	1 major		2 major	1 major	

Abbreviations: ICD, implantable cardioverter‐defibrillator; *N*, number; SVT, supraventricular tachycardia; TWO, *T* wave oversensing; y, year.

^a^ Hybrid system consisting of a subcutaneous defibrillation coil and an epicardial ventricular lead.

^b^ Three patients were switched from a transvenous/hybrid ICD to a fully subcutaneous ICD and in the “total” feature only once.

^c^ One patient experienced shocks with a transvenous ICD and later with a fully subcutaneous ICD and in the “total” features only once.

### 3.1 Initial Implantations

There were a total of 46 initial implantations: 28 transvenous ICDs (smallest patient 14 kg), 15 fully subcutaneous ICDs (S‐ICD; smallest patient 25 kg), two extravascular ICDs (EV‐ICD; smallest patient 58 kg), and one hybrid ICD (combining a subcutaneous intercostal defibrillation coil with an epicardial ventricular lead; smallest patient 21 kg).

The median number of initial implantations was 2.5/year (range 1–7), with a slight upward trend observed over the study period. Based on these data, we estimate that a new indication for ICD arises in approximately 1/22000 children. This estimate was calculated for the 0–17‐year age group to avoid any possible overlap with adult implantation facilities and assumes an annual birth rate in Slovakia of approximately 55 000.

The median age of patients at the time of ICD initial implantation was 14.9 years (3.7–18.9 years). The most frequent underlying diagnoses were long QT syndrome (LQTS), affecting 32% of patients, and hypertrophic cardiomyopathy (HCM), affecting 22% (Table 2).

**TABLE 2:** ICD indications, follow‐up, and mortality.

	Primary prevention	Secondary prevention	
*N* of patients	10 (20%)	39 (80%)	
Patient‐years	27.5	179.7	
Median follow‐up	2 years (1–7)	5 years (0.1–14)	
Mortality	2 (20%)	1 (3%)	
Presenting symptom (*N* of patients)	Heart failure in patients with structural heart disease (7); asymptomatic (3)	VT/VF (32)	Transient loss of consciousness with CPCR (7)
Diagnosis (*N* of patients)	HCM (7); DCM (1); AS (1); Brugada syndrome (1)	LQTS (11); CPVT (6); ARVC (4); HCM (3); DCM (2); SQTS (1); Afib^a^ (1); ccTGA+VSD (1); DTGA+VSD (1); unidentified (2)	LQTS (5); HCM (1); epilepsy^b^ (1)

Abbreviations: Afib, atrial fibrillation; ARVC, arrhythmogenic right ventricular cardiomyopathy; AS, aortic valve stenosis; ccTGA, congenitally corrected transposition of the great arteries; CPCR, cardiopulmonary cerebral resuscitation; DCM, dilated cardiomyopathy; DTGA, dextro‐transposition of the great arteries; HCM, hypertrophic cardiomyopathy; LQTS, long QT syndrome; *N*, number; SQTS, short QT syndrome; VF, ventricular fibrillation; VSD, ventricular septal defect; VT, ventricular tachycardia.

^a^ Patient with atrial fibrillation with rapid atrioventricular conduction, proposed theory was Afib degeneration into ventricular fibrillation.

^b^ Initially with suspicion of Brugada syndrome.

### 3.2 Generator Replacements

A total of seven generator replacements were performed. Three transvenous ICD generators were replaced because of battery depletion in patients with a history of shock therapy, with a median device longevity of 6.5 years (range 4.9–8.9 years). Two transvenous ICD systems were converted to S‐ICDs because of defibrillation lead malfunction. One hybrid ICD underwent generator replacement due to battery depletion (longevity 5.5 years), with concomitant replacement of the subcutaneous intercostal defibrillation coil because of growth‐related malfunction. Another hybrid ICD was converted to an S‐ICD due to defibrillation lead malfunction secondary to patient growth.

### 3.3 Lead Extractions

All lead extractions were uncomplicated and performed using a simple traction technique. During conversion to S‐ICD systems, transvenous defibrillation leads were extracted in both patients with transvenous ICDs, whereas in one patient with a hybrid system both leads were left in situ and only the generator was explanted. In addition, one subcutaneous intercostal defibrillation coil, one transvenous defibrillation lead, and one transvenous atrial lead were extracted for indications unrelated to system conversion.

### 3.4 Summary of All ICD‐Related Complications

A total of 18 ICD‐related complications (lead‐related and non–lead‐related combined) were identified in 12 devices, representing 23% of all implanted devices. Multiple complications occurring within the same device were common. Of these:

- Nine devices (17%) had major complications, comprising three early and six late events.
- Seven devices (13%) had nine minor complications, all of which were early.

In the subcategory of S‐ICDs, all implantations were uneventful, and no complications apart from inappropriate shocks were observed (section 3.5.2 Inappropriate shocks).

#### 3.4.1 Lead‐Related ICD Complications

Major lead‐related complications occurred in 6 devices (11% of all devices). These included (early vs. late complications):

- Four transvenous defibrillation leads: rapid rise in pacing thresholds (late; late), lead fracture (late), and reel syndrome (late).
- One subcutaneous intercostal defibrillation coil affected by growth‐related tension (late).
- One transvenous atrial lead dislodgement in a patient for whom atrioventricular synchrony was clinically important (early).

Minor lead‐related complications were identified in 3 devices (6% of all devices), all of which were early complications:

- One small defibrillator lead thrombus with spontaneous resolution on follow‐up echocardiography.
- One atrial lead sensing malfunction.
- One undefined technical issue necessitating the opening of a second transvenous defibrillation lead during the initial implantation.

#### 3.4.2 Non‐Lead‐Related ICD Complications

Major non–lead‐related complications occurred in 3 devices (6% of all devices). These comprised (early vs. late complications):

- One pocket hematoma requiring aspiration (early).
- One wound infection necessitating 6 weeks of intravenous meropenem, fosfomycin, and fluconazole treatment (early).
- One EV‐ICD wound complication requiring surgical revision due to suspected infection (late).

Minor non–lead‐related complications occurred in 6 devices (11% of all devices), all of which were early complications:

- Three cases of fluidothorax.
- Two peri‐implant atrial fibrillation episodes (both in the same patient with a single‐chamber ICD).
- One superficial wound infection.

### 3.5 ICD Therapy

During the observation period (2012–2025; 207 patient‐years), 20 patients (41%) experienced at least one shock, whereas the remaining 29 patients had no shocks (Table 1). A total of 83 shocks were recorded: 49 (59%) were appropriate, including 32 effective and 17 ineffective shocks, while 34 shocks (41%) were classified as inappropriate. Nine patients (18%) experienced exclusively appropriate shocks, seven patients (14%) experienced exclusively inappropriate shocks, and four patients (8%) experienced both appropriate and inappropriate shocks.

The highest number of effective appropriate shocks (9×) was observed in a patient with LQTS type 3. This LQTS type 3 patient also experienced the highest number of inappropriate shocks (8×) and the highest number of shocks in a single patient (21×). The highest number of ineffective appropriate shocks (6×) was observed in two patients with catecholaminergic polymorphic ventricular tachycardia.

Antitachycardia pacing (ATP) during capacitor charging was activated in all patients and proved effective on three occasions in two individuals: one with suspected short QT syndrome and another with ventricular tachycardia associated with congenital heart disease.

#### 3.5.1 Appropriate Shocks

**FIGURE 1:**
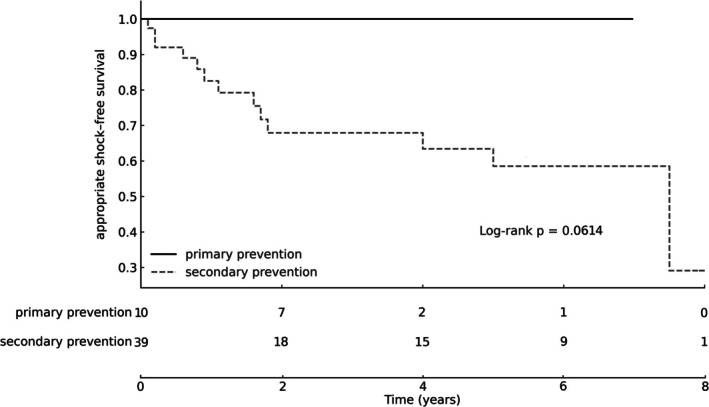
Kaplan–Meier estimates of appropriate ICD shock–free survival in primary and secondary prevention, with numbers at risk displayed at predefined intervals.

Kaplan–Meier analysis demonstrated no appropriate shocks in the primary‐prevention group during follow‐up, whereas shock‐free survival declined over time in the secondary‐prevention group, with an estimated 5‐year shock‐free survival of 59%. No statistically significant difference between groups was observed (log‐rank *χ* ^2^ = 3.50, *p* = 0.061; Table 1, Figure 1).

The most frequent causes of ineffective appropriate shocks were arrhythmia reinitiation in patients with catecholaminergic polymorphic ventricular tachycardia and malfunction of hybrid ICDs related to patient growth.

#### 3.5.2 Inappropriate Shocks

**FIGURE 2:**
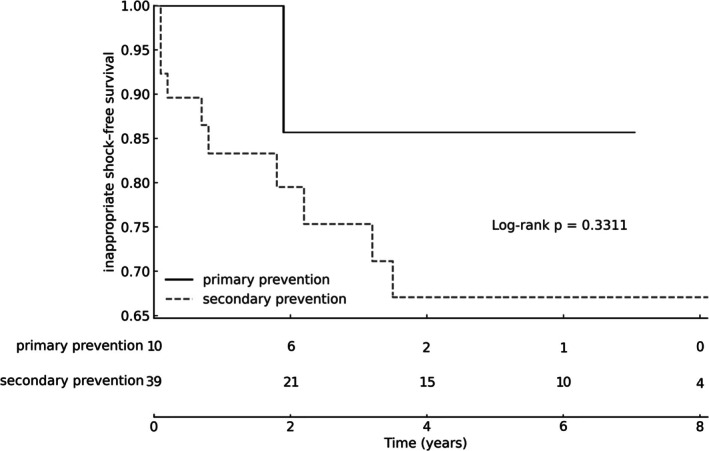
Kaplan–Meier estimates of inappropriate ICD shock–free survival in primary and secondary prevention, with numbers at risk displayed at predefined intervals.

Kaplan–Meier curves for inappropriate shock–free survival showed no significant difference between primary and secondary prevention groups (log‐rank *χ* ^2^ = 0.94, *p* = 0.331). The estimated 5‐year inappropriate shock–free survival in the secondary‐prevention group was approximately 67%, with broadly similar survival patterns observed across both groups during follow‐up (Table 1, Figure 2).

The causes of inappropriate shocks included atrial tachycardia (four episodes), sinus tachycardia during exercise (three episodes), T‐wave oversensing during tachycardia (three episodes), atrial fibrillation with rapid atrioventricular conduction (two episodes), atrial flutter with rapid atrioventricular conduction (one episode), transvenous defibrillation lead fracture (one episode), S‐ICD header air bubbles in the early post‐implantation phase (one episode), and myopotential interference in one S‐ICD system (Table 1).

### 3.6 Mortality and Heart Transplant Endpoints

Three patients (6%) died during follow‐up; none of the deaths were directly related to ICD implantation. Two deaths occurred in patients with Danon disease‐associated HCM, and one in a patient with complex congenitally corrected transposition of the great arteries.

**FIGURE 3:**
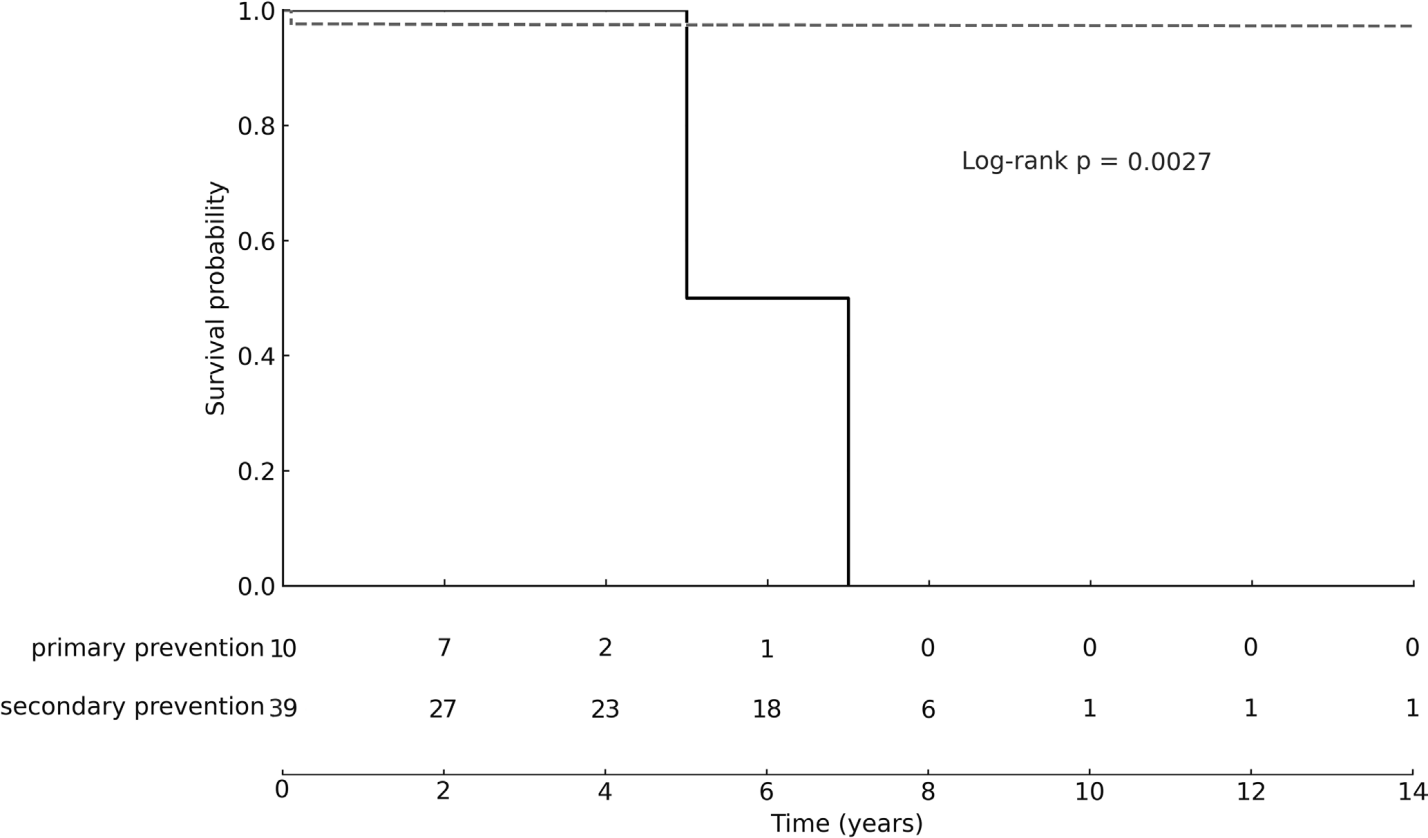
Kaplan–Meier survival analysis of all‐cause mortality in primary and secondary prevention groups, with numbers at risk displayed at predefined intervals.

Kaplan–Meier analysis demonstrated a statistically significant difference in survival between groups (log‐rank *χ* ^2^ = 9.40, *p* = 0.003), with higher observed mortality in the primary prevention group (Table 2, Figure 3). One patient in the primary prevention group underwent heart transplantation. Estimated death‐ or transplant‐free survival at 3 and 5 years was 90% and 45%, respectively, in the primary prevention group, compared with 97% at both time points in the secondary prevention group.

## 4 Discussion

Published data demonstrate a gradual increase in ICD implantation in pediatric populations, predominantly driven by primary prevention indications extrapolated from adult guidelines. This trend is supported by improved survival of patients with congenital heart disease into adulthood, broader availability of genetic testing with increasing diagnostic yield, and enhanced recognition of sudden cardiac death survivors through widespread use of automated external defibrillators. Despite these developments, the overall incidence of pediatric ICD implantation remains low [1, 2, 3, 5, 8, 9, 10].

The estimated incidence of pediatric ICD implantation in Slovakia (1/22000 live births) occupies an intermediate position internationally, being substantially lower than that reported in the United States (~1/7200 in years 2013–2016) and higher than that observed in Japan (~1/50000 in years 2006–2016) [2, 11]. The highly centralized structure of pediatric arrhythmia care in Slovakia provides a unique framework for studying rare interventions such as ICD implantation in children. This national referral model facilitates near‐complete case capture and uniform clinical decision‐making, thereby enabling relatively precise estimates of incidence and clinical course. At the same time, such centralization may favor a more conservative approach to primary prevention ICD implantation, contributing to the predominance of secondary prevention indications observed in our cohort.

### 4.1 Appropriate ICD Therapies

A key finding of the present study is that no appropriate ICD therapies occurred in the primary prevention group during follow‐up. In contrast, Kaplan–Meier analysis showed a progressive decline in shock‐free survival among patients implanted for secondary prevention, with an estimated 5‐year shock‐free survival of 59%. Although no statistically significant difference between groups was observed (log‐rank *χ* ^2^ = 3.50, *p* = 0.061), the divergence in shock‐free survival trajectories is clinically meaningful and reflects the distinct risk profiles of the two populations.

Although the median follow‐up duration in the primary prevention subgroup was relatively short (~2 years), the absence of appropriate ICD therapies is consistent with previous reports demonstrating low rates of appropriate intervention in pediatric primary prevention cohorts. Together, these findings underscore the ongoing uncertainty surrounding the predictive value of adult‐derived risk stratification tools (such as ventricular systolic function and composite risk scores) when applied to children and adolescents. This uncertainty is further illustrated by recent data from Wilkin et al., who demonstrated imperfect discrimination of both the HCM Risk‐Kids and PRIMaCY scores in children with HCM, with predicted risks tending to be overestimated when assessed at a single time point. Although periodic reassessment substantially improved predictive performance, the number of ICDs required to prevent a single arrhythmic event remained high, particularly early after implantation. By contrast, the role of ICD therapy in secondary prevention is well established, with broad consensus supporting implantation in survivors of aborted sudden cardiac death or in patients with documented malignant ventricular arrhythmias or arrhythmia‐related syncope [8, 9, 12, 13]. This clear risk–benefit profile is reflected in our cohort, in which secondary prevention accounted for 80% of ICD implantations and in which appropriate shocks, observed in 27% of patients (comparable to the 22%–61% reported in the literature), occurred exclusively in this group, further reinforcing the robustness of the indication [1, 3, 14, 15, 16, 17, 18, 19, 20].

### 4.2 Inappropriate Shocks and Device Programming

Inappropriate shocks occurred in 22% of patients, consistent with previously reported rates of 21%–29% [1, 3, 14, 15, 16, 17, 18, 19, 20]. Kaplan–Meier analysis demonstrated no significant difference in inappropriate shock–free survival between primary and secondary prevention groups. The causes of inappropriate shocks were predominantly supraventricular tachyarrhythmias and sensing‐related issues, including atrial tachycardia, sinus tachycardia during exercise, T‐wave oversensing, lead fracture, S‐ICD header air bubbles in the early post‐implantation phase, and myopotential interference.

Importantly, a marked reduction in inappropriate shocks was observed following implementation of contemporary ICD programming strategies. The most substantial programming changes for transvenous ICDs were introduced between 2013 and 2014 and included elimination of ventricular tachycardia treatment zones in patients with cardiac channelopathies, use of higher ventricular fibrillation detection thresholds (initially 220–230 bpm, later 230–250 bpm), and prolonged detection durations [8, 20, 21, 22, 23, 24, 25] These adjustments resulted in a rapid decline in inappropriate shocks without loss of sensitivity for the treatment of life‐threatening ventricular arrhythmias. These findings emphasize the need for a holistic approach to ICD management in children, incorporating lifestyle counseling, optimization of pharmacotherapy, and individualized device programming to minimize inappropriate therapies while preserving clinical efficacy. Physically active patients, particularly competitive athletes, remain the most challenging subgroup, owing to lower adherence to medical therapy and a higher incidence of sinus tachycardia during peak exercise.

### 4.3 Device‐Related Complications

Device‐related complications remain a significant drawback of ICD therapy in children. In our cohort, 18 ICD‐related complications were identified in 23% of implanted devices, with major complications affecting 17% of devices. This incidence is comparable to published pediatric series reporting major complication rates of 10%–26% and up to 41% over long‐term follow‐up. Multiple complications per device were common, underscoring the cumulative procedural burden associated with lifelong device therapy initiated in childhood. Lead‐related complications accounted for the majority of major events, particularly late complications such as lead fracture, reel syndrome, pacing threshold rise, and growth‐related lead tension. These findings are consistent with previous studies demonstrating that ICD recipients experience higher complication rates than pacemaker recipients, largely due to system complexity and the mechanical vulnerability of defibrillation leads in growing, active patients [3, 4, 6, 7, 10, 14, 26, 27].

In contrast, all S‐ICD implantations in our cohort were uneventful, and no device‐related complications other than inappropriate shocks were observed. This favorable acute and mid‐term safety profile supports accumulating evidence that S‐ICDs represent an attractive alternative for pediatric patients without pacing requirements. Consistent with international trends, we also observed a gradual shift from transvenous ICDs to S‐ICDs over the study period, likely driven by the desire to reduce lead‐related morbidity [1, 5].

### 4.4 Extravascular ICDs

Experience with EV‐ICDs in our cohort remains limited. Two highly active adolescents underwent EV‐ICD implantation to provide additional protection of the defibrillation lead via a substernal position, particularly where ATP was considered potentially beneficial. Implantation and sensing parameters were satisfactory. However, follow‐up was extremely short (1.2 patient‐years), and one patient required surgical revision for local infection. These findings should therefore be interpreted as demonstrating procedural feasibility rather than long‐term efficacy or safety, and overinterpretation should be avoided.

### 4.5 Mortality and Transplant Outcomes

Overall mortality in the present cohort was 6%, which falls within the range reported in the literature (2.9%–8.3%). All deaths were related to underlying structural heart disease, and no peri‐implantation or ICD‐related deaths were observed, consistent with previously reported rates of 0%–2.7% [1, 3, 4, 6, 28]. No deaths occurred in patients with cardiac channelopathies.

Kaplan–Meier analysis demonstrated significantly higher mortality in the primary prevention group (log‐rank *χ* ^2^ = 9.40, *p* = 0.003), with one patient requiring heart transplantation, despite the absence of appropriate ICD therapies in this subgroup. These findings suggest that progressive heart failure or nonarrhythmic disease progression may represent a dominant competing risk in selected pediatric primary prevention patients, potentially limiting the protective impact of ICD therapy against sudden arrhythmic death alone.

### 4.6 Future Directions

Finally, the integration of genetic testing into routine clinical practice is likely to play an increasingly important role in refining risk stratification. In Slovakia, the introduction of systematic genetic autopsy in 2024 represents a critical step towards improved identification of inherited arrhythmia syndromes, enhanced cascade screening, and more personalized ICD decision‐making. Such approaches may ultimately help balance the competing risks of sudden arrhythmic death and device‐related morbidity in pediatric patients.

### 4.7 Limitations

This study has a retrospective, single‐center design. The median follow‐up duration was relatively short, and the overall cohort size was limited, particularly in the primary prevention and EV‐ICD subgroups. Consequently, the statistical power of the analyses is restricted, and the findings should be interpreted with caution. Furthermore, patients aged exactly 18 years who were never evaluated at the Pediatric Cardiac Center may have undergone ICD implantation at an adult‐care facility; if such patients existed, their number was likely small.

## 5 Conclusion

In this nationwide, single‐center cohort, pediatric ICD implantation in Slovakia occurred at an estimated incidence of approximately 1/22000 live births and was predominantly performed for secondary prevention, with appropriate ICD therapies observed exclusively in this group. Notably, no appropriate therapies occurred in primary prevention patients, despite higher overall mortality, underscoring ongoing uncertainty regarding primary prevention indications in children and the impact of competing nonarrhythmic risks. Device‐related adverse events, including inappropriate shocks (22%) and major complications (17%), were frequent, emphasizing the substantial long‐term burden of ICD therapy initiated in childhood. In this context, S‐ICDs demonstrated a favorable short‐ to mid‐term safety profile in patients without pacing requirements, although longer follow‐up is required to define their role in pediatric practice. Together, these findings emphasize the need for pediatric‐specific, dynamic risk stratification strategies and individualized ICD management to optimize patient selection and minimize device‐related morbidity.

## Author Contributions

Marko Bjeloševič: concept, data analysis, drafting article, approval of article. Peter Olejník: data analysis, statistics, critical revision of article, approval of article. Jaroslav Tomko: statistics, data collection, drafting article, approval of article. Michal Chalupka: statistics, data collection, drafting article, approval of article. Viera Illíková: concept, data analysis, critical revision of article, approval of article.

## Funding

The authors have nothing to report.

## Conflicts of Interest

The authors declare no conflicts of interest.

## Data Availability

The data that support the findings of this study are available on request from the corresponding author. The data are not publicly available due to privacy or ethical restrictions.

## References

[1] H. Mori , N. Sumitomo , K. Tsutsui , et al., “Efficacy of SubcutAneous Implantable cardioVErter‐Defibrillators in ≤ 18 Year‐Old CHILDREN: SAVE‐CHILDREN Registry,” International Journal of Cardiology 15, no. 371 (2023): 204–210. 10.1016/j.ijcard.2022.09.008 36087632

[2] S. Baskar , H. Bao , K. E. Minges , D. S. Spar , and R. J. Czosek , “Characteristics and Outcomes of Pediatric Patients Who Undergo Placement of Implantable Cardioverter Defibrillators: Insights From the National Cardiovascular Data Registry,” Circulation. Arrhythmia and Electrophysiology 11, no. 9 (2018): e006542. 30354291 10.1161/CIRCEP.118.006542

[3] G. Frommeyer , S. Feder , M. Bettin , et al., “Long‐Term Single‐Center Experience of Defibrillator Therapy in Children and Adolescents,” International Journal of Cardiology 15, no. 271 (2018): 105–108. 10.1016/j.ijcard.2018.05.130 29885825

[4] J. Thuraiaiyah , B. T. Philbert , A. S. Jensen , et al., “Implantable Cardioverter Defibrillator Therapy in Paediatric Patients for Primary vs. Secondary Prevention,” Europace 26, no. 9 (2024): euae245. 39345160 10.1093/europace/euae245 PMC11440178

[5] P. Wieniawski , M. Buczyński , M. Grabowski , J. Winter , and B. Werner , “Subcutaneous Implantable Cardioverter Defibrillators for the Prevention of Sudden Cardiac Death: Pediatric Single‐Center Experience,” International Journal of Environmental Research and Public Health 19, no. 18 (2022): 11661. 36141934 10.3390/ijerph191811661 PMC9517274

[6] M. Lewandowski , P. Syska , I. Kowalik , et al., “Fifteen Years' Experience of Implantable Cardioverter Defibrillator in Children and Young Adults: Mortality and Complications Study,” Pediatrics International 60, no. 10 (2018): 923–930. 29998526 10.1111/ped.13660

[7] J. Atallah , C. C. Erickson , F. Cecchin , et al., “Multi‐Institutional Study of Implantable Defibrillator Lead Performance in Children and Young Adults: Results of the Pediatric Lead Extractability and Survival Evaluation (PLEASE) Study,” Circulation 127, no. 24 (2013): 2393–2402. 23694966 10.1161/CIRCULATIONAHA.112.001120

[8] K. Zeppenfeld , J. Tfelt‐Hansen , M. De Riva , et al., “2022 ESC Guidelines for the Management of Patients With Ventricular Arrhythmias and the Prevention of Sudden Cardiac Death: Developed by the Task Force for the Management of Patients With Ventricular Arrhythmias and the Prevention of Sudden Cardiac Death of the European Society of Cardiology (ESC) Endorsed by the Association for European Paediatric and Congenital Cardiology (AEPC),” European Heart Journal 43, no. 40 (2022): 3997–4126.36017572

[9] M. J. Shah , M. J. Silka , J. N. A. Silva , et al., “2021 PACES Expert Consensus Statement on the Indications and Management of Cardiovascular Implantable Electronic Devices in Pediatric Patients,” Heart Rhythm 18, no. 11 (2021): 1888–1924. 34363988 10.1016/j.hrthm.2021.07.038

[10] K. M. Burns , F. Evans , and J. R. Kaltman , “Pediatric ICD Utilization in the United States From 1997‐2006,” Heart Rhythm 8, no. 1 (2011): 23–28. 20887811 10.1016/j.hrthm.2010.09.073 PMC3010480

[11] H. Asakai , A. Shimizu , T. Mitsuhashi , et al., “Current Trends in Implantable Cardioverter‐Defibrillator Therapy in Children—Results From the JCDTR Database,” Circulation Journal 83, no. 1 (2018): 52–55. 30344201 10.1253/circj.CJ-18-0712

[12] J. Brugada , N. Blom , G. Sarquella‐Brugada , et al., “Pharmacological and Non‐Pharmacological Therapy for Arrhythmias in the Pediatric Population: EHRA and AEPC‐Arrhythmia Working Group Joint Consensus Statement,” Europace 15, no. 9 (2013): 1337–1382. 23851511 10.1093/europace/eut082

[13] M. Wilkin , D. Khraiche , E. Panaioli , et al., “Independent External Evaluation of Pediatric Hypertrophic Cardiomyopathy Risk Scores in Predicting Severe Ventricular Arrhythmias,” Circulation. Arrhythmia and Electrophysiology 18, no. 3 (2025): e012932. 39973620 10.1161/CIRCEP.124.012932

[14] B. J. Maron , P. Spirito , M. J. Ackerman , et al., “Prevention of Sudden Cardiac Death With Implantable Cardioverter‐Defibrillators in Children and Adolescents With Hypertrophic Cardiomyopathy,” Journal of the American College of Cardiology 61, no. 14 (2013): 1527–1535. 23500286 10.1016/j.jacc.2013.01.037

[15] D. Lawrence , N. Von Bergen , I. H. Law , et al., “Inappropriate ICD Discharges in Single‐Chamber Versus Dual‐Chamber Devices in the Pediatric and Young Adult Population,” Journal of Cardiovascular Electrophysiology 20, no. 3 (2009): 287–290. 19175843 10.1111/j.1540-8167.2008.01322.x

[16] J. H. M. Heersche , N. A. Blom , F. van de Heuvel , et al., “Implantable Cardioverter Defibrillator Therapy for Prevention of Sudden Cardiac Death in Children in The Netherlands,” Pacing and Clinical Electrophysiology 33, no. 2 (2010): 179–185. 20025697 10.1111/j.1540-8159.2009.02603.x

[17] S. P. Etheridge , S. Sanatani , M. I. Cohen , C. A. Albaro , E. V. Saarel , and D. J. Bradley , “Long QT Syndrome in Children in the Era of Implantable Defibrillators,” Journal of the American College of Cardiology 50, no. 14 (2007): 1335–1340. 17903632 10.1016/j.jacc.2007.05.042

[18] A. Çeliker , H. Olgun , T. Karagoz , S. Özer , S. Özkutlu , and D. Alehan , “Midterm Experience With Implantable Cardioverter‐Defibrillators in Children and Young Adults,” Europace 12, no. 12 (2010): 1732–1738. 20852288 10.1093/europace/euq340

[19] C. I. Berul , G. F. Van Hare , N. J. Kertesz , et al., “Results of a Multicenter Retrospective Implantable Cardioverter‐Defibrillator Registry of Pediatric and Congenital Heart Disease Patients,” Journal of the American College of Cardiology 51, no. 17 (2008): 1685–1691. 18436121 10.1016/j.jacc.2008.01.033

[20] L. D. D. Petersen , M. K. Christiansen , L. N. Pedersen , J. C. Nielsen , A. K. Broendberg , and H. K. Jensen , “Implantable Cardioverter‐Defibrillator Therapy and Device‐Related Complications in Young Patients With Inherited Cardiomyopathies or Channelopathies: A 17‐Year Cohort Study,” Europace 20, no. 11 (2018): 1849–1855. 29697814 10.1093/europace/euy081

[21] C. Y. Miyake , G. Webster , R. J. Czosek , et al., “Efficacy of Implantable Cardioverter Defibrillators in Young Patients With Catecholaminergic Polymorphic Ventricular Tachycardia: Success Depends on Substrate,” Circulation. Arrhythmia and Electrophysiology 6, no. 3 (2013): 579–587. 23667268 10.1161/CIRCEP.113.000170

[22] B. L. Wilkoff , L. Fauchier , M. K. Stiles , et al., “2015 HRS/EHRA/APHRS/SOLAECE Expert Consensus Statement on Optimal Implantable Cardioverter‐Defibrillator Programming and Testing,” in Europace: European Pacing, Arrhythmias, and Cardiac Electrophysiology: Journal of the Working Groups on Cardiac Pacing, Arrhythmias, and Cardiac Cellular Electrophysiology of the European Society of Cardiology, vol. 18 (Oxford University Press, 2016), 159–183. 26585598 10.1093/europace/euv411

[23] M. K. Stiles , L. Fauchier , C. A. Morillo , and B. L. Wilkoff , “2019 HRS/EHRA/APHRS/LAHRS Focused Update to 2015 Expert Consensus Statement on Optimal Implantable Cardioverter‐Defibrillator Programming and Testing,” Heart Rhythm 17, no. 1 (2020): e220–e228. 31103461 10.1016/j.hrthm.2019.02.034

[24] D. Jain , T. Prendiville , S. Adel , D. Ward , and D. Crinion , “7 Channelopathies and Implantable Cardioverter Defibrillator Therapy: A Specialist Centre's Experience,” BMJ 108 (2022): 1–A7.

[25] F. Roses‐Noguer , J. W. E. Jarman , J. R. Clague , and J. Till , “Outcomes of Defibrillator Therapy in Catecholaminergic Polymorphic Ventricular Tachycardia,” Heart Rhythm 11, no. 1 (2014): 58–66. 24120999 10.1016/j.hrthm.2013.10.027

[26] R. J. Czosek , K. Meganathan , J. B. Anderson , T. K. Knilans , B. S. Marino , and P. C. Heaton , “Cardiac Rhythm Devices in the Pediatric Population: Utilization and Complications,” Heart Rhythm 9, no. 2 (2012): 199–208. 21907171 10.1016/j.hrthm.2011.09.004

[27] C. M. Janson , A. R. Patel , W. J. Bonney , K. Smoots , and M. J. Shah , “Implantable Cardioverter‐Defibrillator Lead Failure in Children and Young Adults: A Matter of Lead Diameter or Lead Design?,” Journal of the American College of Cardiology 63, no. 2 (2014): 133–140. 24140671 10.1016/j.jacc.2013.09.033

[28] D. U. Chung , M. Hochadel , J. Senges , et al., “Procedural Outcome and 1‐Year Follow‐Up of Young Patients Undergoing Implantable Cardioverter‐Defibrillator Implantation‐Insights From the German DEVICE I+II Registry,” Journal of Clinical Medicine 13, no. 13 (2024): 3858. 38999424 10.3390/jcm13133858 PMC11242717

